# Antibody-Free Labeling of Malaria-Derived Extracellular Vesicles Using Flow Cytometry

**DOI:** 10.3390/biomedicines8050098

**Published:** 2020-04-27

**Authors:** Elya Dekel, Paula Abou Karam, Yael Ohana-Daniel, Mirit Biton, Neta Regev-Rudzki, Ziv Porat

**Affiliations:** 1 Faculty of Biochemistry, Department of Biomolecular Sciences, Weizmann Institute of Science, Rehovot 7610001, Israel; elya.dekel@weizmann.ac.il (E.D.); paula.aboukaram@weizmann.ac.il (P.A.K.); yaelimeli@gmail.com (Y.O.-D.); mirit.biton@weizmann.ac.il (M.B.); 2 Flow Cytometry Unit, Life Sciences Core Facilities, Weizmann Institute of Science, Rehovot 7610001, Israel

**Keywords:** malaria, extracellular vesicles, *Plasmodium falciparum*, flow cytometry

## Abstract

Extracellular vesicles (EVs) are cell-derived membrane-bound structures that are believed to play a major role in intercellular communication by allowing cells to exchange proteins and genetic cargo between them. In particular, pathogens, such as the malaria parasite *Plasmodium (P.)* *falciparum*, utilize EVs to promote their growth and to alter their host’s response. Thus, better characterization of these secreted organelles will enhance our understanding of the cellular processes that govern EVs’ biology and pathological functions. Here we present a method that utilizes a high-end flow cytometer system to characterize small EVs, i.e., with a diameter less than 200 nm. Using this method, we could evaluate different parasite-derived EV populations according to their distinct cargo by using antibody-free labeling. It further allows to closely monitor a sub-population of vesicles carrying parasitic DNA cargo. This ability paves the way to conducting a more ‘educated’ analysis of the various EV cargo components.

## 1. Introduction

Extracellular vesicles (EVs) are a group of cell-secreted vesicles that either originate from the endosomal system (as in the case of exosomes) or shed from the plasma membrane (in the case of microvesicles). These vesicles are present in biological fluids and are involved in multiple physiological and pathological processes [1,2,3,4]. EVs carry diverse content, such as RNA [5,6], DNA [7,8,9], proteins [10,11] and lipids [12,13], which they transfer to specific target cells, making them a key player in intercellular communication [14]. Specifically, EVs from *P. falciparum*-iRBCs deliver different active parasitic cargo components [9,15,16,17,18] and play a role in host manipulation [9]. The 48-h asexual blood stage of *P. falciparum* is cyclic and involves differentiation from invading merozoite to ring, to trophozoite and to mature schizont, which then rupture and release daughter cells, merozoites, which go on to invade naïve RBCs [19] (Supplementary Figure S1A). It has been recently shown that these parasites secrete EVs during the ring-stage that contain parasitic genomic DNA [9,16,18], which EVs from later stages (i.e., trophozoite and schizont) most probably lack. Thus, it is becoming clear that there are several subsets of secreted EVs, each harboring different cargo components [20]. However, in this expanding field, much remains unknown regarding the biogenesis of the vesicle secretion pathway, the EV’s uptake by target cells and the EV’s cellular roles [21], as well as the specific role of each EV subpopulation. Even though new technologies [22] are beginning to explore and characterize sub-populations of EVs, there is still a lack of knowledge, which highlights the need to improve the identification of surface markers on individual EV particles.

Numerous methods are used to study EVs, ranging from bulk characterization of the cargo components (e.g., high-throughput analyses of protein, DNA and RNA profiles [20,23]) to single particle analysis methods showing particle size and distribution or morphology (e.g., nanoparticle tracking analysis [NTA]) [24,25], electron microscopy, atomic force microscopy and flow cytometry) [26,27,28,29,30,31,32,33]. Among these tools, flow cytometry is one of the main methods to evaluate the identity of multiple surface EV markers on individual particles. In this technology, particles in suspension flow through a chamber, where they are illuminated by a set of lasers. The parameters that can be collected are light scattering, gathered either at a low angle (0–5^°^) off the particles (forward scatter, FSC) or at an angle of approximately 90 degrees (side scatter, SSC). If the particles are fluorescently labeled, the emission can be detected by a set of photomultiplier tubes (PMTs) after the light passes through a set of spectral filters. This allows to acquire information on each individual particle’s light-scattering properties and obtain multiple fluorescence measurements, up to 30 parameters in the high-end instruments. However, this technology is optimized for cells rather than small particles, as the limit of detection for membrane-bound EVs is usually above 0.5 μm [34]. This restraint is due to the light scatter profile of small particles, the size of the laser beam, the sensitivity of the detectors and the level of the noise. In addition, EVs can be detected by Imaging Flow Cytometry, which utilizes a sensitive CCD camera [9,16,34,35,36], but the collection rate is much slower and the number of collected channels is limited to 10. Thus, identifying EVs by flow cytometry is a challenging task. One way to overcome these limitations is to attach the EVs to larger particles, which are easier to detect. This approach, however, does not enable the analysis of individual particles [37,38].

In an effort to overcome these limitations, we utilized the Bio-Rad ZE5™ Cell Analyzer (Bio-Rad, Hercules, CA, USA) to analyze the subpopulations of *P. falciparum*-derived EVs and measure the presence of the DNA cargo within the subgroups of parasitic EVs. One of the challenges in studying malaria-derived EVs is the lack of commercially available antibodies specific for malaria-derived antigens. To overcome this, the method we developed allows us to label EVs without the need for antibodies but by labeling other cellular components. Additionally, the ZE5 flow cytometer has several advantages in terms of analyzing small particles. It is equipped with a forward scatter detector optimized for small particles, which utilizes a high-power, 100 mW, 405 nm laser [31,39,40]. In addition, it uses distilled water as the sheath fluid, which we further filter using a 0.025 μm filter (Merck-Millipore, Burlington, MA, USA) to minimize the background particles.

## 2. Materials and Methods

### 2.1. Parasite Line and Culture

The NF54 parasite line was obtained from the Malaria Research Reference Reagent Resource Center (MR4, Manassas, VA, USA). Parasites were maintained in culture in A+ erythrocytes at 4% hematocrit in RPMI-HEPES supplemented with 0.5% (w/v) AlbumaxII (Invitrogen, Waltham, MA, USA), as previously described [41].

### 2.2. EV Isolation and Fluorescence Staining

EVs were isolated from the NF54 strain in a highly parasitemic (approximately 8%) *P. falciparum*-infected red blood cell (iRBC) culture using an OPTIMA90X ultracentrifuge (Beckman, Pasadena, CA, USA) equipped with a TI70 rotor, as previously described [42]. Briefly, 200 mL of a parasite growth medium was collected and then cellular debris was removed by centrifugation at 413 × *g* for 5 min, 1650 × *g* for 10 min (5804 Centrifuge, Eppendorf, Hamburg, Germany), followed by centrifugation at 10,000 rpm for 1 h in an RC5C PLUS (Sorvall, Waltham, MA, USA) with a SLA-1500 rotor. The supernatant was filtered in a 0.45 µm filter and concentrated down to a 10 mL volume using a VivaCell 100,000 MWCO PES (Sartorious Staedium, Goettingen,). The resultant medium was centrifuged at 150,000 × *g* for 18 h to pellet EVs. The pellet was resuspended in PBS−/−, and the purified EVs were stained according to the manufacturer’s protocol with slight modifications, as described below. We used several fluorescent stains for the different vesicle compounds: 5 µM Hoechst (HO) 33342 dye (Invitrogen, Waltham, MA, USA) for DNA; 1 mg/mL thiazole orange (TO, Sigma Aldrich, St. Louis, MO, USA) for RNA cargo; 5 nM CFSE (Sigma Aldrich St. Louis, MO, USA ) for protein cargo; and 5 µM PKH26 dye (Sigma Aldrich Israel) for lipid cargo. CFSE and Hoechst stains were incubated with *P. falciparum*-derived EVs at 37 °C at a 1:1 *v*/*v* ratio and reached 2.5 nM and 2.5 µM, respectively. PKH26 was prepared according to the manufacturer’s protocol and was resuspended with equal volumes of EV solution. TO-labeled EVs were incubated with EVs at a 1 µL/mL ratio at 37 °C for 30 min. They were then washed in ice-cold PBS and precipitated again in an ultracentrifuge at 150,000 × *g* for 18 h. Next, the EV pellet was washed and resuspended in PBS−/−, and the size and concentration of the labeled EVs were measured using a NanoSight NS300 instrument (Malvern Instruments Ltd., Worcestershire, United Kingdom) with the associated laser [16,24].

### 2.3. Nanoparticle Tracking Analysis

Nanoparticle tracking analysis (NTA, Nanosight NS300) was performed at 20 °C. Sample size distributions were obtained in a liquid suspension (1:1000 dilution in PBS−/−) by the analysis of Brownian motion via light scattering. The camera level was set to 13 and the gain to 1, laser 405 nm or 488 nm without filter.

### 2.4. EV Uptake into Monocytes

Monocyte cells of the THP1 cell line were cultured [43] overnight in RPMI1640+ l-glutamine (Biological Industries Ltd., Beit Ha’Emek, Israel) and 10% FBS (Biological Industries Ltd.). Prior to the vesicle treatment, cells were washed in PBS−/−, resuspended in RPMI1640+ (Biological Industries Ltd.) and plated in a 6-well plate, ~1.5 × 10^6^ cells per well. THP1 cells were incubated with labeled *P. falciparum*-iRBC-derived EVs (at a concentration of 8 × 10^11^ par/mL) for 5 min before being washed in PBS and analyzed by IFC or ZE5.

### 2.5. Multispectral IFC Analysis

Cells were imaged using a multispectral IFC (ImageStreamX mark II, Amnis Corp., Seattle, WA, USA, part of Luminex Ltd.). In the EV uptake measurements, EVs were labeled and ~1.5 × 10^6^ EVs were imaged using the IFC. The ImageStreamX uses calibration beads that are 3 μm in diameter. To exclude these beads from the acquisition, objects were gated according to their area and intensity of the side scatter channel (Ch06), and the uniform bead population was easily identified and eliminated. At least 5 × 10^4^ cells were collected from each sample and the data were analyzed using the manufacturer’s image analysis software (IDEAS 6.2; Amnis Corp.). Images were compensated for fluorescent dye overlap by using single-stain controls. THP1 cells were gated for single cells, using the area and aspect-ratio features, and for focused cells, using the Gradient RMS feature, as previously described [44]. Cropped cells were further eliminated by plotting the cell area of the bright field image against the Centroid X feature (the number of pixels in the horizontal axis from the left corner of the image to the center of the cell mask). EV internalization was evaluated using several features, including the intensity (the sum of the background − subtracted pixel values within the masked area of the image) and max pixel (the largest value of the background subtracted pixel within the image mask).

### 2.6. ZE5 Analysis

EVs were analyzed using the Bio-Rad ZE5™ Cell Analyzer (Bio-Rad). The machine ran with distilled water as the sheath fluid, which was further filtered by a 0.025 μm filter (Merck-Millipore), followed by its washing to clean residual particles in the tubes before each experiment. The machine’s parameters and settings are described in detail in Supplementary Table S1. EVs were labeled as described above.

## 3. Results

### 3.1. Internalization of Pf-Derived EVs by THP1 Cells

To test the ability of the ZE5 to adequately detect the fluorescent signal of the exosomes, we first assessed its ability to detect the internalization of *P. falciparum*-derived EVs by immune cells, such as monocytes (THP1 cell line), in an established experimental system. To evaluate the quality of our results, we compared the ZE5 output to that of the ImageStream Imaging Flow Cytometer [16]. Briefly, the EV uptake experiment entailed staining the *P. falciparum*-derived EVs with thiazole orange (TO), an RNA dye, and introducing them to THP1 cells stained with the Hoechst (HO) nuclear marker for 5 min. Following the EV incubation period, THP1 cells were analyzed using either the ImageStream Flow Cytometry or the ZE5 analyzer (Supplementary Figure S1B). Both detection methods exhibited similar results: The uptake percentage was 99% for the TO-positive signal inside HO-labeled THP1 cells (Supplementary Figure S1C).

### 3.2. Detection of Nanoparticles Using the ZE5 Analyzer and NTA Analysis

**Figure 1:**
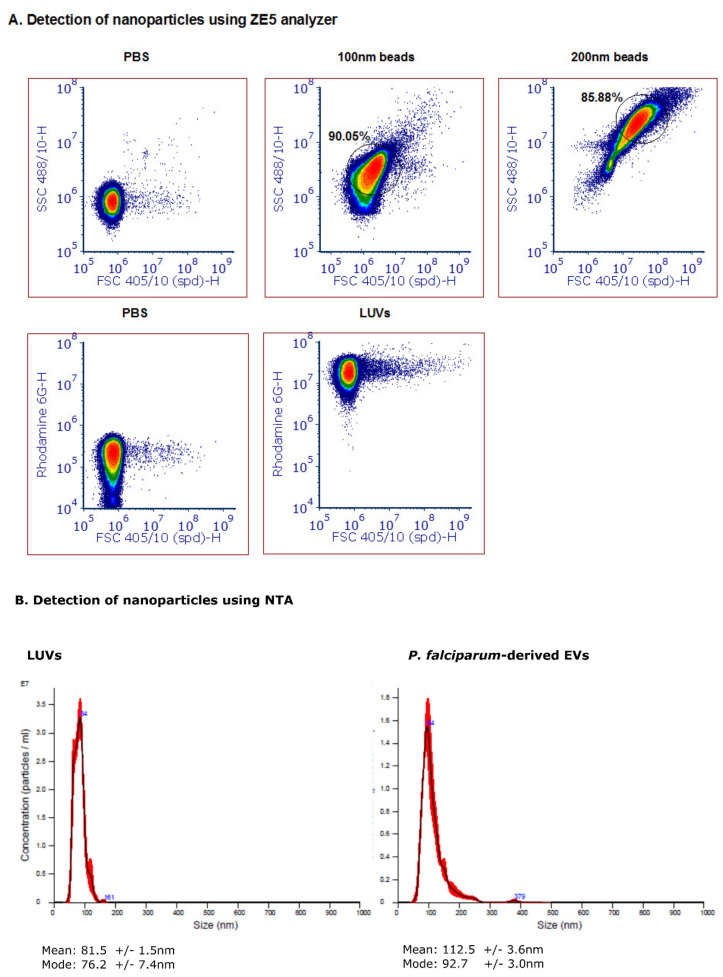
Detection of nanoparticles using the ZE5 analyzer and NTA analysis. (**A**) Polystyrene beads of 200 nm (top row, middle panel) and 100 nm (top row, right panel) in diameter were gated according to FSC 405/10 and SSC 488/10 light scattering. The analysis was visualized with a density plot and the gating was performed according to the main population. Rhodamine-PE large unilamellar vesicles (LUVs) were detected according to FSC 405/10 and the Rhodamine distribution. A PBS sample was used as control for undetected particles. (**B**) Nanoparticle tracking analysis (NTA) (Malvern Instruments Ltd., Nanosight NS300) was performed at 20 °C on Rho-LUVs and EVs to determine the concentration and size. Sample size distributions were calibrated in a liquid suspension (1:1000 dilution) by the analysis of Brownian motion via light scattering. The camera level was set to 13 and the gain to 1, laser 405 nm or 488 nm without filter.

We next examined the ability of the 405 nm laser detector of the cell analyzer to detect free nanoparticles in solution, namely, to differentiate between two synthetic particles: polystyrene latex beads of 100 and 200 nm in diameter (Figure 1A). A clear separation was seen between the two populations of the synthetic beads, and over the background noise. However, since the polystyrene beads have a higher refractive index than membrane-bound vesicles, the FSC and SSC values are very different (up to two orders of magnitude lower) [34]. We, therefore, also examined Large Unilamellar Vesicles (LUVs), i.e., liposomes of uniform size distribution and staining (Figure 1A, bottom panel). rhodamine-PE LUVs (phosphatidylethanolamine conjugated with rhodamine) were freshly prepared, as previously described [45]. In brief, a mixture of egg phosphatidylcholine, cholesterol and sphingomyelin at a 1:1:1 ratio (Sigma Chemical Israel) was inserted into a dried film of lipid mixture. Next, it was suspended in PBS and subjected to several cycles of freezing–thawing and then extrusion through polycarbonate membranes with a pore size of 1 μm and a diameter of 0.1 µm. The obtained LUVs were then compared to the malaria-derived EVs harvested from a high-parasitemia (approximately 8%) *P. falciparum*-iRBC culture through a series of ultracentrifugation steps [42]. Even though the FSC values did not significantly differ between the background noise of the PBS and the EVs, the fluorescent dye labeling showed a clear separation between the labeled EVs and the background. Our Nanoparticle Tracking Analysis (NTA) [24] demonstrated a similar size distribution of the LUVs and the EVs, between 50-120 nm (Figure 1B).

### 3.3. Detection of Different Types and Amounts of Pf-Derived EV Cargo Using the ZE5 Analyzer

**Figure 2:**
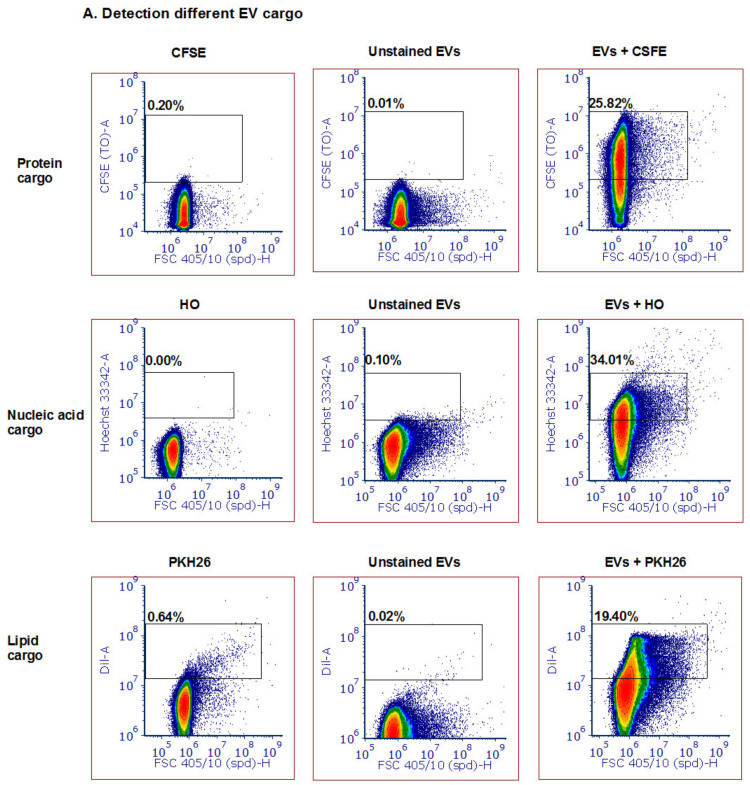

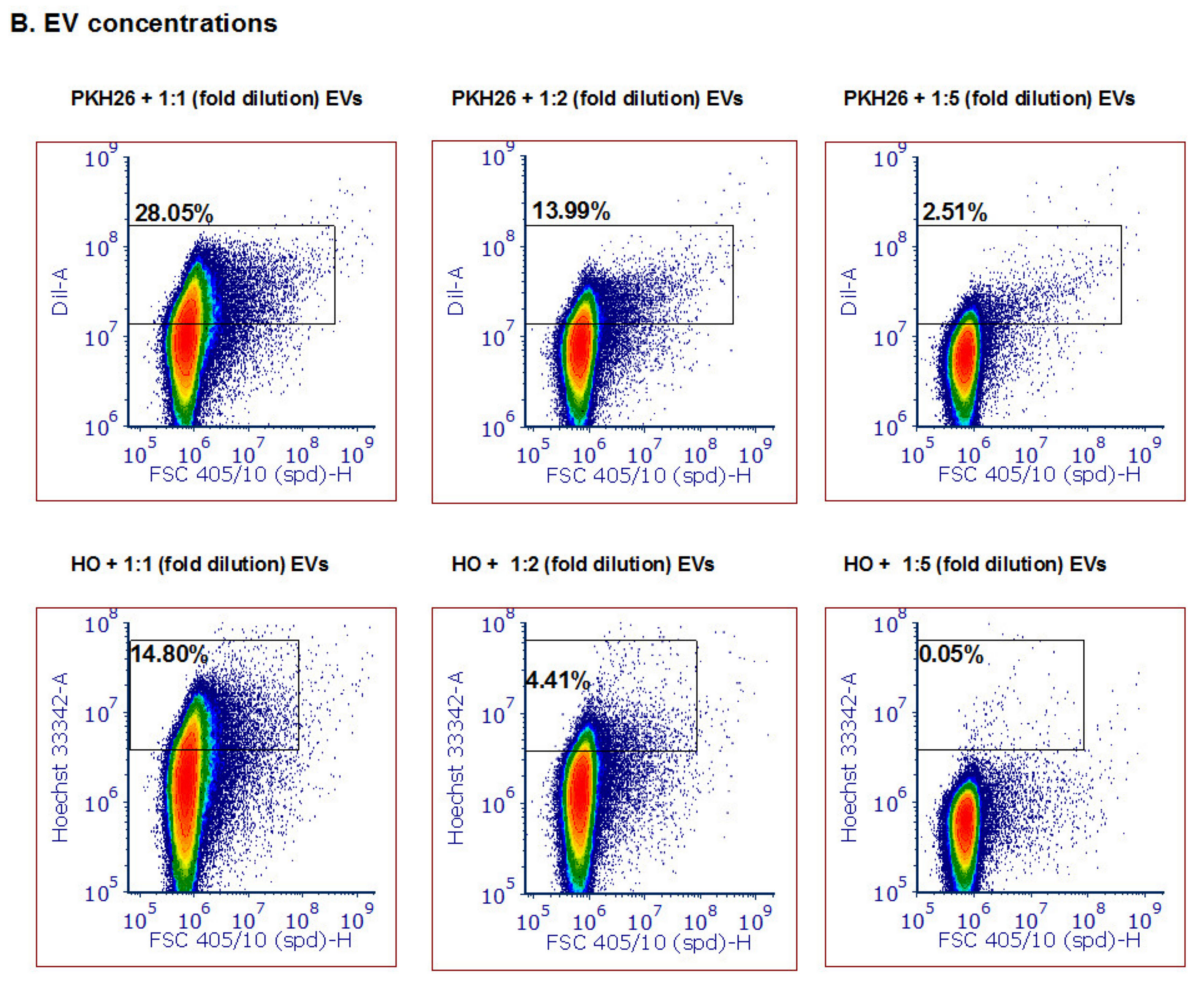
Detection of different types and amounts of *Pf*-derived EV cargo using the ZE5 analyzer. (**A**) *Pf*-derived EVs were stained using three different dyes to visualize different types of cargo components: proteins with the CFSE marker (top row), nucleic acids with the HO marker (middle row), and lipids with the PKH26 marker (bottom row). As controls, solutions containing free dye or unstained EVs were used. (**B**) Detection of different *Pf*-derived EV concentrations was achieved by diluting the EVs with the same dye (PKH26 and HO) amount. On the left: high concentration (8 × 10^11^ par/mL); in the center and on the right: lower concentrations of EVs.

Following the establishment of the ability to detect small labeled particles using the ZE5™ cell analyzer (measured by other techniques to be the size of about 100 nm), we directed our efforts to examine whether this system can monitor various vesicle cargo components. We used several fluorescent dyes to stain the different EV cargo components: proteins (5 nM CFSE dye, Sigma Aldrich Israel), lipid membranes (5 µM PKH26 dye, Sigma Aldrich Israel) and DNA (5 µM Hoechst 33342 dye, Invitrogen). CFSE and Hoechst stains were incubated with *P. falciparum*-derived EVs at 37 °C at a 1:1 *v*/*v* ratio and reached 2.5 nM and 2.5 µM, respectively. PKH26 was prepared according to the manufacturer’s protocol and was resuspended with equal volumes of EV solution. As seen in Figure 2A, we were able to detect EVs stained by the protein dye [upper panel], DNA dye [middle panel] and lipid dye (bottom panel). As controls, we used unstained EVs and the different dyes without EVs in PBS −/− in order to ensure the signal indeed specifically labels EVs. All the dyed EVs (three different stains) demonstrated higher staining than the controls; the unstained EVs alone and dye solution controls were used to set the background. The results were similar to stained EVs by IFC (Supplementary Figure S3). Furthermore, to validate that the staining is due to the cargo components of the EVs and not due to dye aggregates [46] or unspecific background binding, we added different EV concentrations to equal amounts of the dye (Figure 2B, compare to 1B, left panel). To avoid PKH26 lipid dye aggregates, the staining was performed for only 2 min at 37 °C. The results indeed show that with each dilution of the EVs the dye signal decreases accordingly. In addition, to exclude the possibility that multiple particles were falsely detected as a single particle (also known as “swarm effect”), we performed a two-fold serial dilution of EVs, followed by staining with PKH26 (Supplementary Figure S2A). Although the percentage of positive events (compared with the dye solution control) indeed decreased with the dilutions, the average intensity remained similar (Supplementary Figure S2B).

### 3.4. Detection of Different Subpopulations of Pf-Derived EVs Using the ZE5 Analyzer

**Figure 3:**
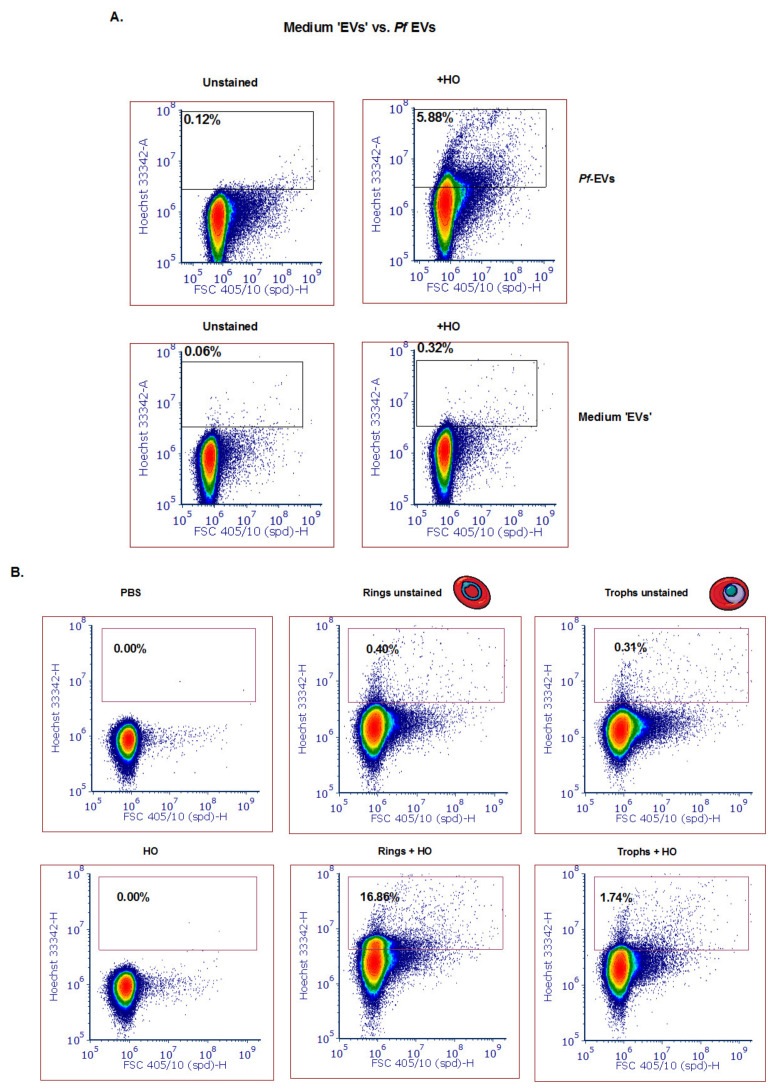
Detection of different subpopulations of *Pf*-derived EVs using the ZE5 analyzer. (**A**) Top row: Mixed population of *Pf*-derived EVs (ring and trophozoite) stained with HO. Gating was done according to the unstained EVs. Bottom row: medium-derived ‘EVs’ were used as a control for absence of DNA content. (**B**) *Pf*-derived EVs from different parasite stages (ring and trophozoite) were stained with HO to visualize the DNA inside the EVs. Gating was done according to the unstained EV population.

To rule out the possibility that the measured signal is due to aggregates of the medium serum supplement (e.g., Albumax II), we examined the content of “medium EVs”, which are produced in the same manner as described above but without the iRBCs. Indeed, a very low DNA signal was detected as compared to the signal from DNA-harboring EVs (Figure 3A). However, the protein dye CFSE produced a signal in the “medium EV” sample, probably because it detected the protein components of the serum (data not shown).

Importantly, we sought to ascertain whether we could apply the cell analyzer to differentiate between ring-stage DNA-containing parasite EVs and trophozoite- or schizont-stage EVs lacking gDNA cargo, as previously shown [18]. To identify *P. falciparum*-derived specific subpopulations of EVs produced at different blood stages, we synchronized the parasites with 5 % sorbitol [41] and collected media after 12 h in order to separate between the ring and trophozoite stage (Supplementary Figure S1A). Following extraction of EVs from the ring and trophozoite stages, we examined their DNA content (Figure 3B). Impressively, the EVs harvested from the culture of the ring-stage parasites showed a positive signal for DNA content that was approximately 15 times more Hoechst positive than that of the trophozoite stage parasite EVs (16.76 % vs. 1.74 % of the EVs were positive for Hoechst, respectively). These results not only support a previous report [9] that the parasite at the ring stage releases EVs harboring DNA, but also demonstrate that the cell-analyzer detector serves as a valid tool to study EV cargo existence.

## 4. Discussion

In this study, we utilized the advantages of the small particle detector and improved fluidics of the ZE5™ flow cytometer in order to detect small particles. Although the shift in FSC is very small, there are other advantages to this method, such as improved fluidics and sensitivity, which facilitate small-particle detection.

EVs were detected without the need for specific antibodies, but rather by specific cargo staining only. The dyes used enable the labeling of unfixed EVs with markers that detect proteins (CFSE), lipids (PKH26) and DNA (Hoechst). The latter demonstrates distinct parasite population detection according to asexual stage, supporting the results obtained from a previous study [9] that showed the co-localization of the DNA with the EVs, suggesting that the detected DNA was internal vesicle cargo, rather than strands stuck to the outside of the vesicles [9].

This method enables to further advance EV research by offering the means for the rapid and better characterization of the cargo components of distinct EV populations. Thus it paves the way to the development of high-throughput EV labeling for specific proteins, and even different species of RNA and DNA sequences.

## 5. Conclusions

- Malaria-derived EVs can be detected by the ZE5™ flow cytometer using antibody-free labeling.
- The DNA cargo within the vesicles secreted from the early developmental blood stage (the ring stage) can be detected.

## Supplementary Materials

The following are available online at https://www.mdpi.com/2227-9059/8/5/98/s1.
