# Supplementary material for: Antibody-Free Labeling of Malaria-Derived Extracellular Vesicles Using Flow Cytometry
Source: Biomedicines. 2020 Apr 27;8(5):98. doi: 10.3390/biomedicines8050098 (PMC7277110; doi:10.3390/biomedicines8050098)
Supplement: Supplementary file 1 [file biomedicines-08-00098-s001.zip › biomedicines-767937 supplementary done/Supplementary files MDPI/Supplamentary figures.docx]

Supplementary figure 1:


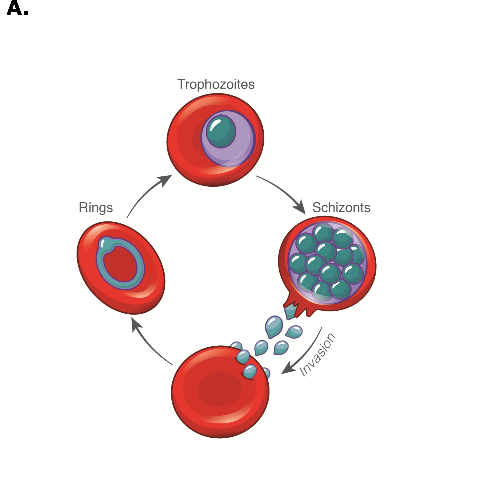


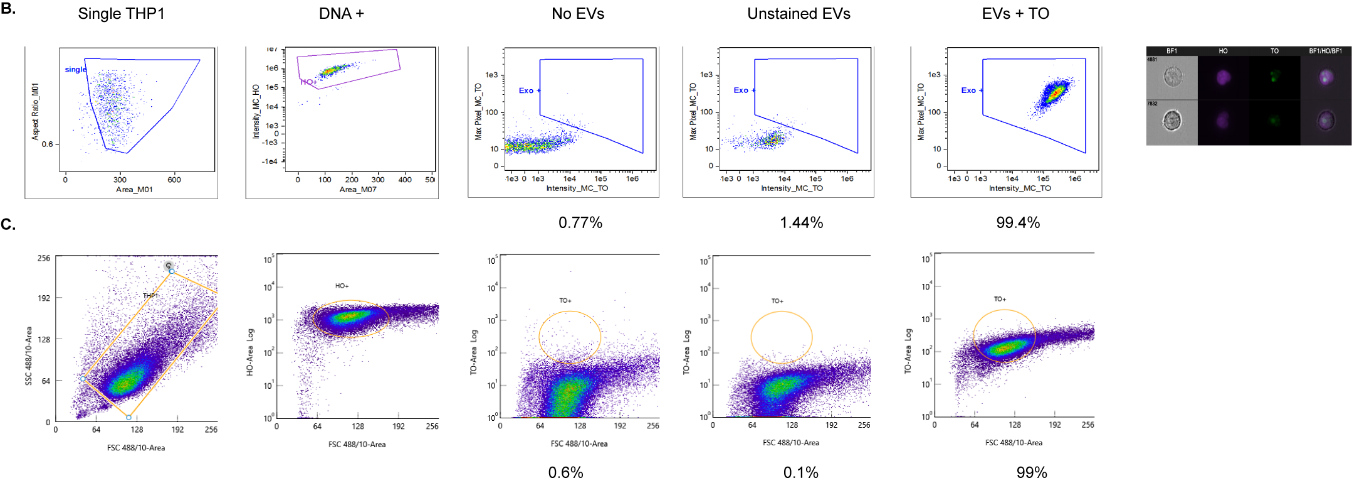


**Supplementary Figure 1: Internalization of *Pf*-derived EVs by THP1 cells**. **[A**.**]** *Pf* asexual blood cycle. Merozoites invade mature RBCs, marking the start of the ring stage, which lasts 16 h, after which the parasites mature into trophozoites, which form schizonts 36 h post invasion. 48 h post invasion, mature schizonts will rupture and release up to 30 merozoites into the bloodstream. Blood-stage parasites are responsible for the clinical manifestations of the disease. **[B.]** Detection of the internalization (uptake) of RNA-stained *Pf*-derived EVs by THP1 cells using ImageStream Flow Cytometry (IFC) [B] or ZE5 [C] analyzer. 1.5 × 10^6^ THP1 cells were live-stained with a DNA dye [Hoechst 33342] and treated with 8 × 10^11^ *Pf*-derived EVs stained with an RNA dye [Thiazole orange (TO)] for 5 min, washed twice with PBS and placed on ice in the dark. Following staining, cells were detected as spots inside recipient cells by an IFC [B] or ZE5 [C] analyzer. Unstained THP1 cells and unstained EVs were used as controls. Gating shows the percentage of TO-positive EVs (99%) internalized by THP1 cells, gated according to unlabeled EVs and ‘free TO’ (referred to as ‘No EVs’), which was also used as a control to exclude the possibility of dye randomly entering cells. Right panel shows examples of THP1 cells with internalized EVs labeled with TO. **[C**.**]** Uptake of RNA-stained *Pf*-derived EVs using Bio-Rad ZE5 analyzer. stained EVs (8×10^11^ ) were introduced into 1.5×10^6^ THP-1 cells for 5 min, washed twice with PBS and placed on ice in the dark. Following staining, cells were detected as spots inside recipient cells by a IFC [B] or ZE5 [C] analyzer. Unstained THP1 cells and unstained EVs were used as controls. Representative results from at least three experiments are shown.

Supplementary figure 2:


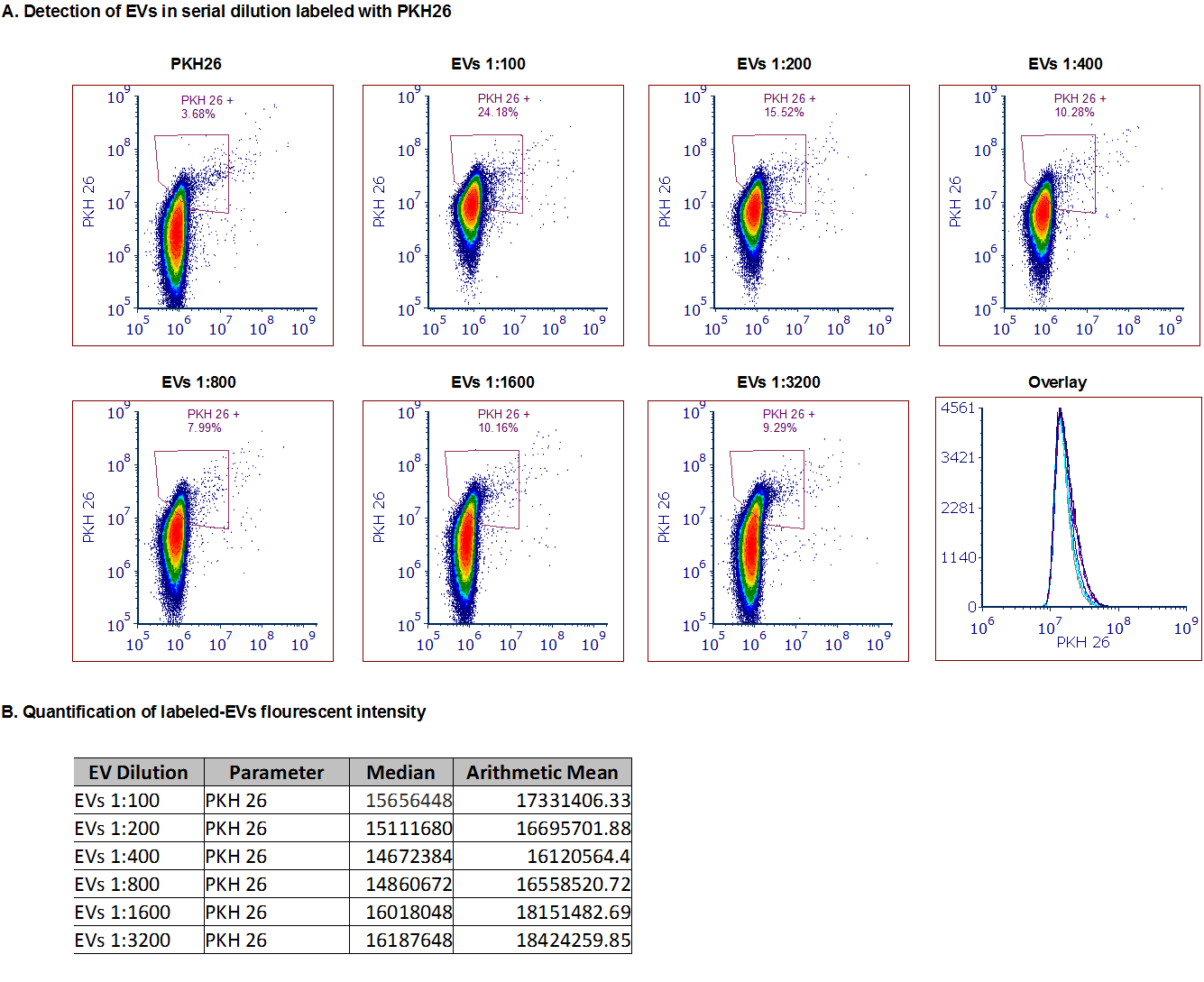


**Supplementary Figure 2: Detection of labeled EVs in serial dilutions.** **[A.]** 5 × 10^11^ EVs were diluted 1:100, followed by five serial dilutions of 1:2. Next, EVs were labeled with equal volumes of PKH26 for 1 min (1:1, v/v) and the stained population was then analyzed by the ZE5 flow cyotmeter. The analysis was visualized with a density plot and the gating was performed according to the free-dye solution. Overlay histogram represents the different EV dilutions according to the dye intensity. **[B.]** Quantification of the fluorescence intensity of PKH26-labeled EVs. Results represents 2 independent biological repeats.

Supplementary figure 3:


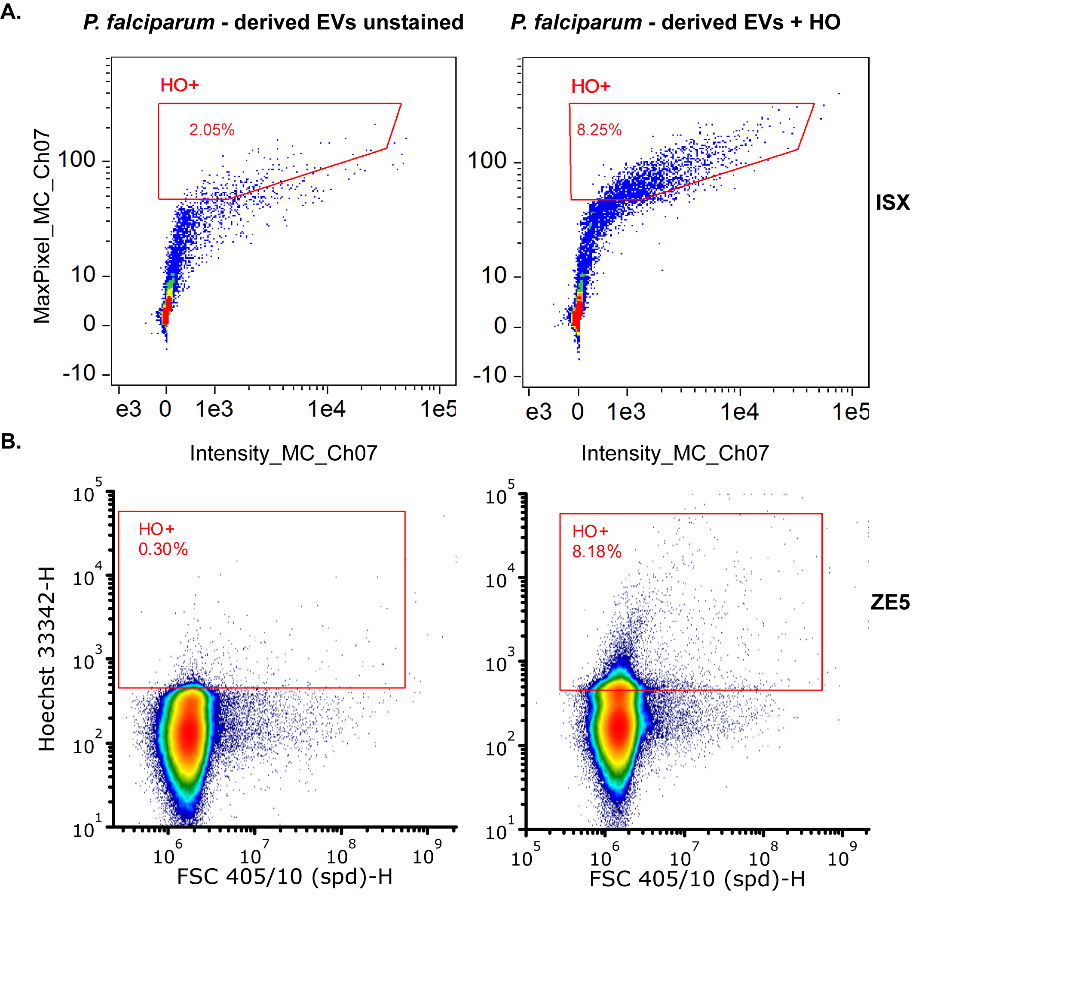


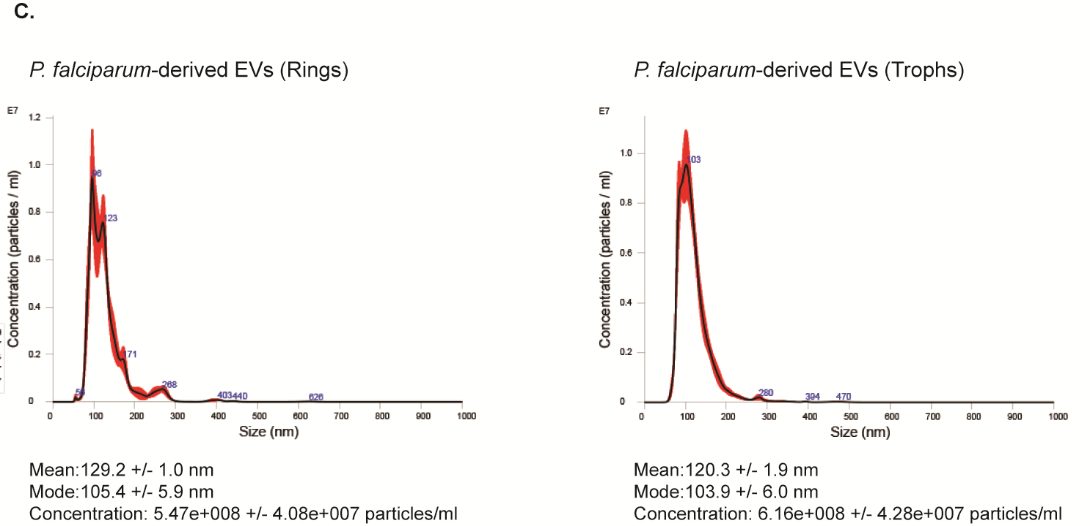


**Supplementary Figure 3: Measurements of *P. falciparum*-derived EVs**. **[A-B.]** *P. falciparum*-derived EVs (from the ring stage) were isolated and analyzed using [A] ImageStream and [B] ZE5. EVs were stained using Hoechst 33342. Unstained EVs were used as controls. **[C.]** NTA analysis of the size and concentration of the isolated EVs from rings (left panel) and trophs (right panel). An equal volume of medium was used to isolate EVs from the two stages.
