# Supplementary material for: Antibody-Free Labeling of Malaria-Derived Extracellular Vesicles Using Flow Cytometry
Source: Biomedicines. 2020 Apr 27;8(5):98. doi: 10.3390/biomedicines8050098 (PMC7277110; doi:10.3390/biomedicines8050098)
Supplement: Supplementary file 1 [file biomedicines-08-00098-s001.zip › biomedicines-767937 supplementary done/Supplementary files MDPI/Supplementary table 1.docx]

| **A.** |  |  |  |  |  |
| --- | --- | --- | --- | --- | --- |
|  | **Category** | **Method description** | **Details** |  |  |
|  | **EV isolation** | Ultracentrifugation | Media was collected and cellular debris removed by centrifugation for 5 min at 1500 r.p.m., 10 min at 3000 r.p.m (5810 R, eppendorf) and 17,000 r.p.m (Sorvall Lynx 4000, Thermo Scientific). The supernatant was concentrated using a VivCell 100,000 MWCO PES (Sartorious Stedium) and centrifuged for 18 hr at 37,000 r.p.m to pellet EVs at 4 °C, in a 70.1 Ti rotor (Optima XE-90, Beckman Coulter, Fullerton, CA, USA). |  |  |
|  | **EV characterization** | Atomic Force Microscopy, Transmission Electron Microscopy, Cryo Electron Microscopy, NanoSight | N. Regev-rudzki et al., “Cell-cell communication between malaria-infected red blood cells via exosome-like vesicles. - PubMed - NCBI,” Cell, vol. 153, no. 5, pp. 1120–1133, 2013. X. Sisquella et al., “Malaria parasite DNA-harbouring vesicles activate cytosolic immune sensors,” Nat. Commun., vol. 8, no. 1, 2017. |  |  |
|  | **EV functional studies** | This work does not involve functional assays |  |  |  |
|  |  |  |  |  |  |
| **B.** |  |  |  |  |  |
|  | **Category** | **Method description** | **Details** | | |
|  | **Daily QC test** | The system acquires the manufacturer QC beads at 500 events per second, than it automatically adjusts laser delays and PMT voltages so that the mean intensity of the bead population falls into data channel 128 in all fluorescence channels. CV increases and PMT voltage changes are compared against the QC criteria. |  | | |
|  | **Daily rainbow beads test** | The Spherotech 8-peak rainbow calibration particles present eight different fluorescent intensities. CV of the highest intensity is lower than 7% in each laser. Bead population is higher than 70%. |  | | |
|  | **Acquisition Parameters** | Flow rate | 0.1 ul/s | | |
|  |  | Events | 1000000 | | |
|  |  | Sample acquired volume | 0.27-0.29 ul | | |
|  |  | Trigger/Threshold | FSC 405/10 0.02% | | |
|  |  |  | SSC 488/10 0.03% | | |
|  | **Lasers** |  | **Filter Set** | **Name/Fluorophore** | **PMT Voltage** |
|  |  | 355 nm, 50 mW | 447/60 | Hoechst 33342 | 520 |
|  |  | 405 nm, 100 mW | 405/10 | FSC | 389 |
|  |  | 488 nm, 100 mW | 488/10 | SSC | 538 |
|  |  |  | 525/35 | CFSE/Thiazole Orange /PKH 26 | 500 |
|  |  | 561 nm, 50 mW | 577/15 | Dil | 813 |
|  |  |  | 589/15 | PE - Rhodamin | 595 |
