# Supplementary figures and images for: Antibody-Free Labeling of Malaria-Derived Extracellular Vesicles Using Flow Cytometry
Source: Biomedicines. 2020 Apr 27;8(5):98. doi: 10.3390/biomedicines8050098 (PMC7277110; doi:10.3390/biomedicines8050098)

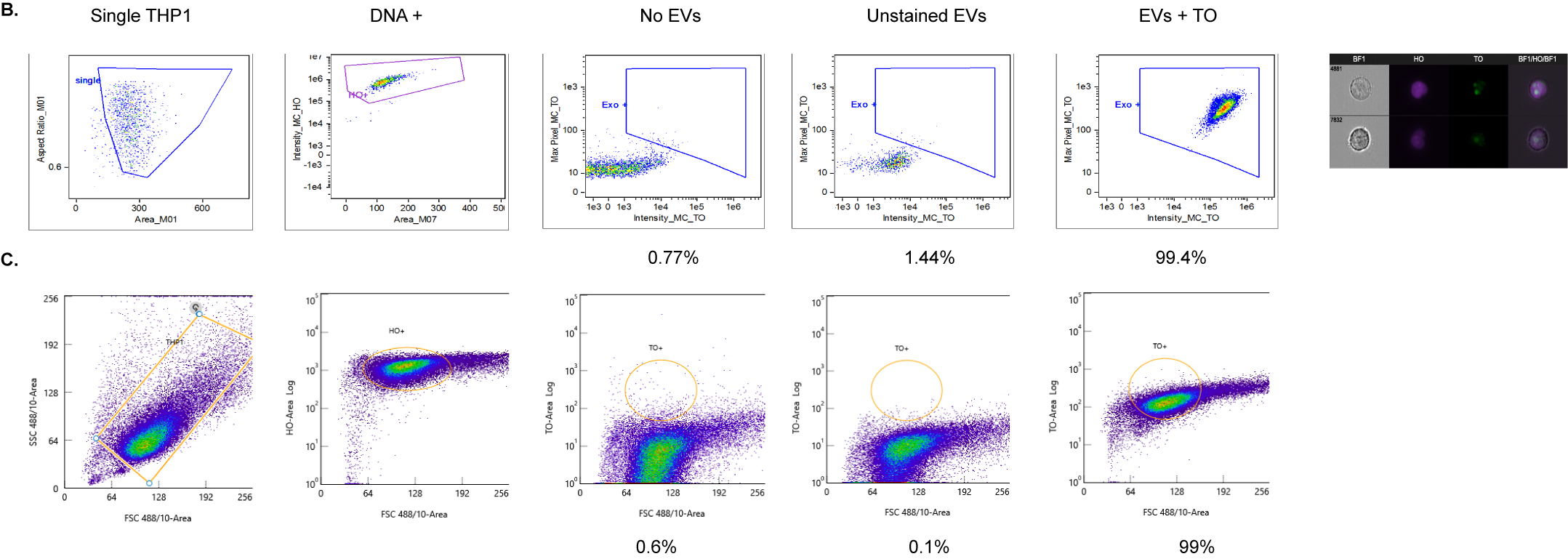

Supplement: Supplementary file 1 [file biomedicines-08-00098-s001.zip › biomedicines-767937 supplementary done/Supplementary files MDPI/Supp figure 1B,C aiv5.tif]

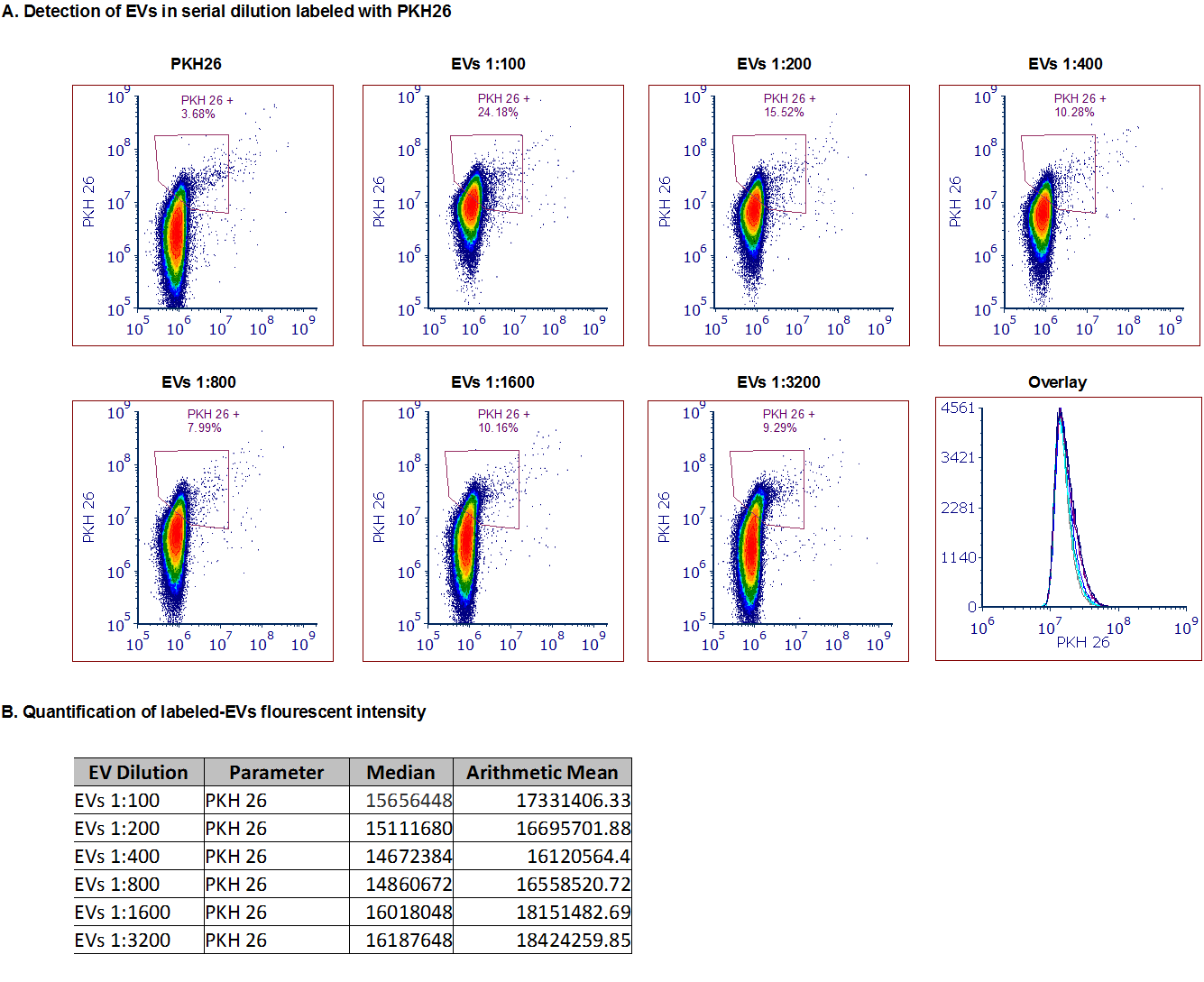

Supplement: Supplementary file 1 [file biomedicines-08-00098-s001.zip › biomedicines-767937 supplementary done/Supplementary files MDPI/Supp Figure 2A,B v4.tif]

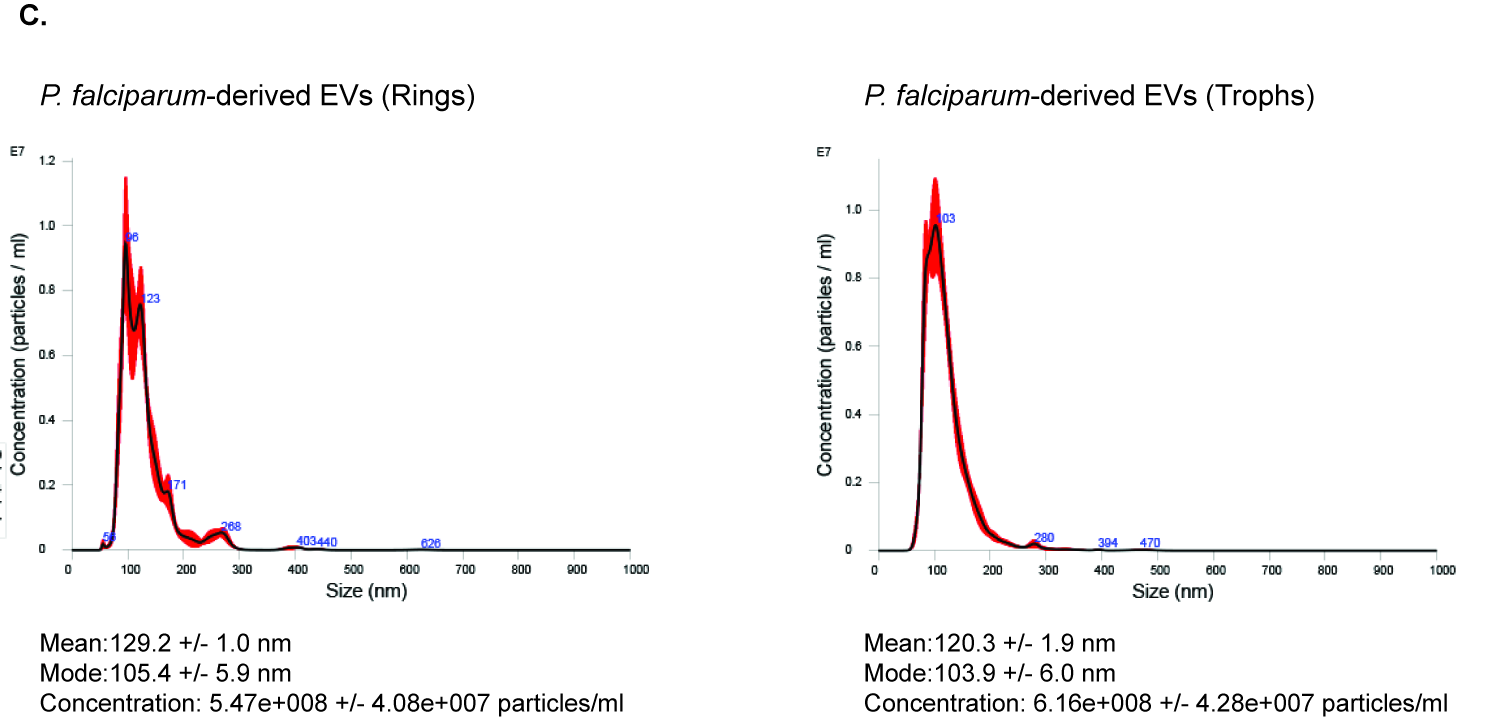

Supplement: Supplementary file 1 [file biomedicines-08-00098-s001.zip › biomedicines-767937 supplementary done/Supplementary files MDPI/Supp. fig 3C. Comparison between rings and trophs concentrationsv2.tif]

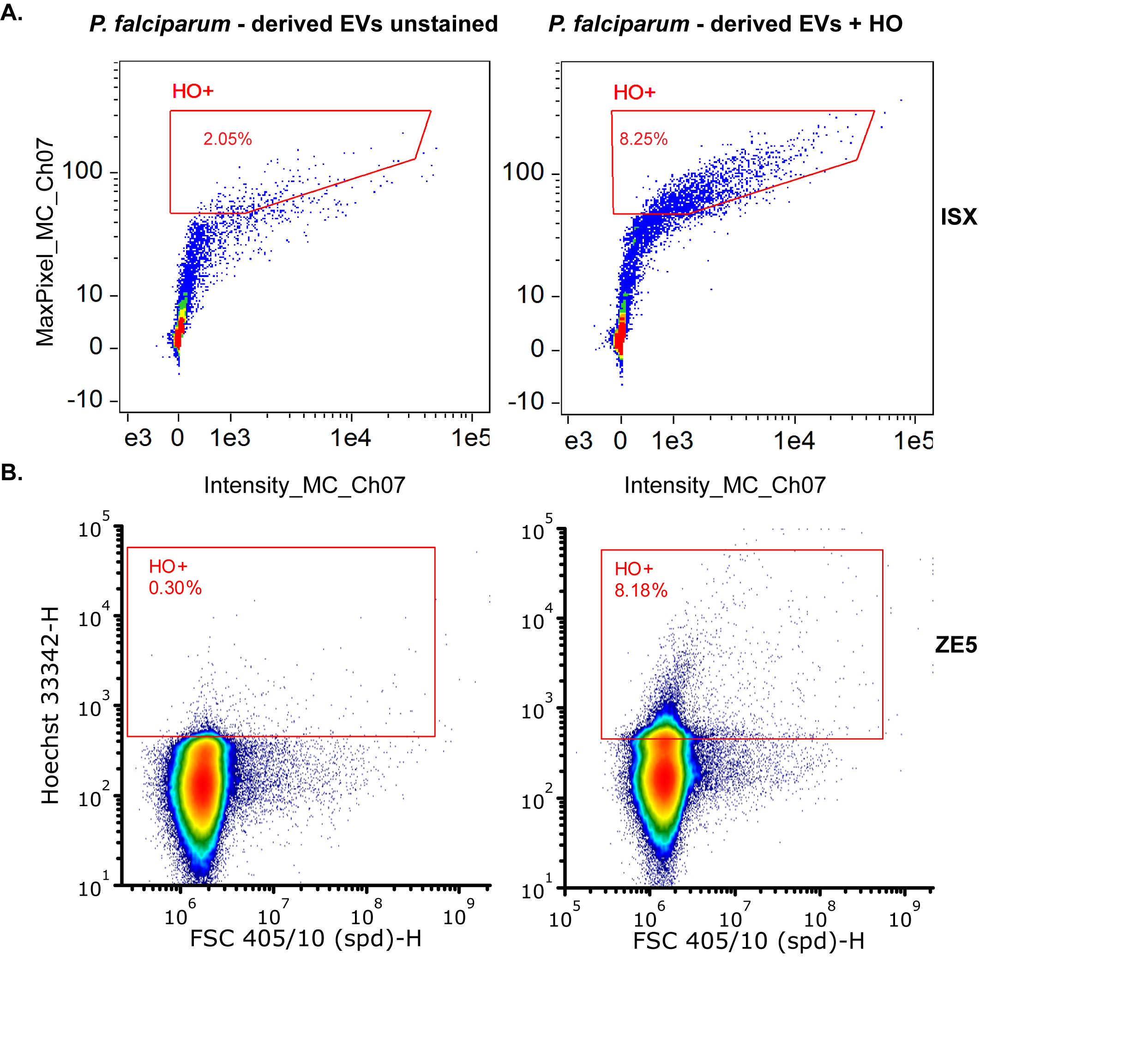

Supplement: Supplementary file 1 [file biomedicines-08-00098-s001.zip › biomedicines-767937 supplementary done/Supplementary files MDPI/Supp. figure 3A,B ISX vs ze5 EVsv2.tif]

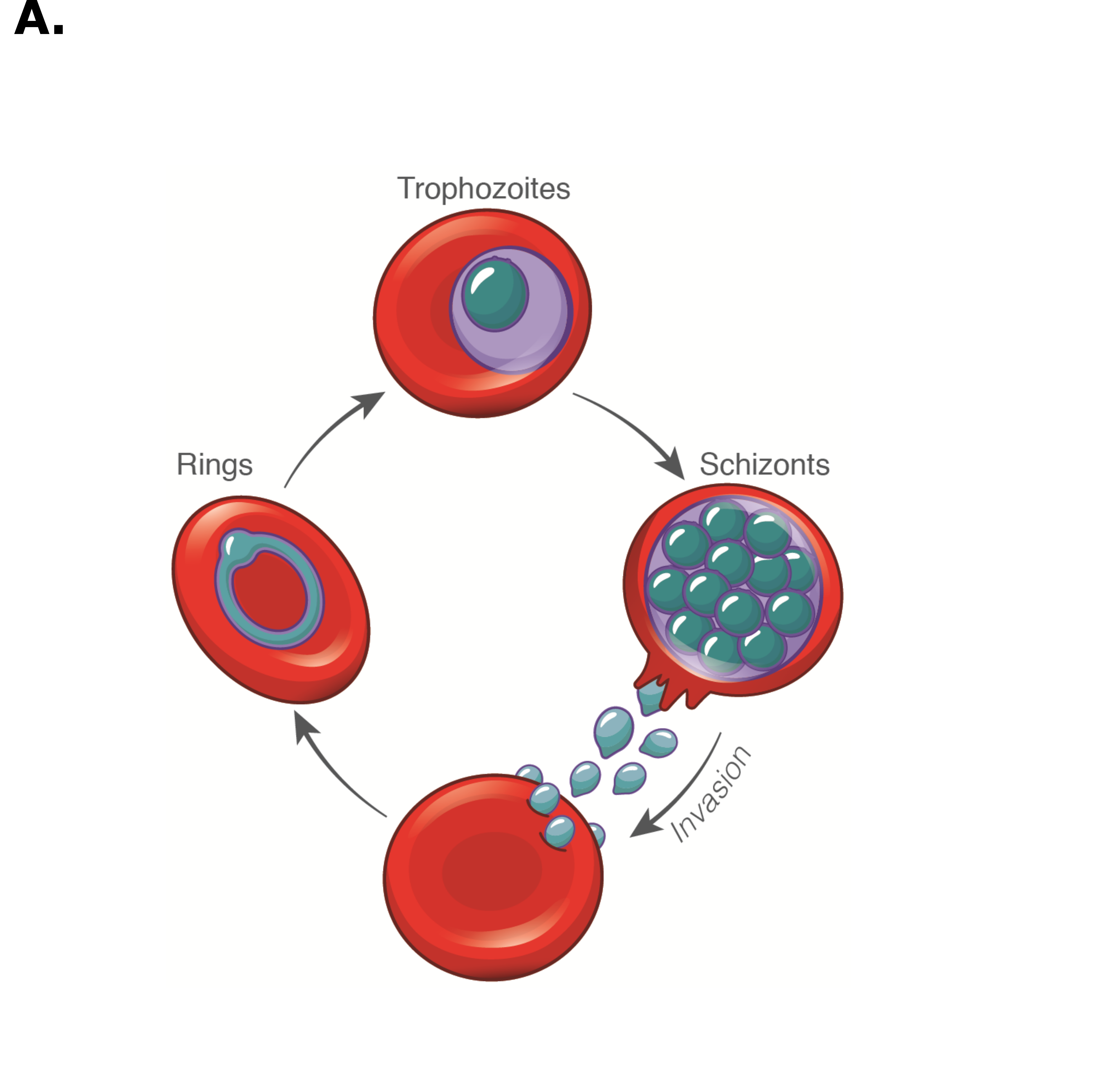

Supplement: Supplementary file 1 [file biomedicines-08-00098-s001.zip › biomedicines-767937 supplementary done/Supplementary files MDPI/Supplamentary figure 1A v2.tif]
